# Mechanistic Understanding of the Antiviral Properties of Pistachios and Zeaxanthin against HSV-1

**DOI:** 10.3390/v15081651

**Published:** 2023-07-29

**Authors:** Rosamaria Pennisi, Paola Trischitta, Maria Pia Tamburello, Davide Barreca, Giuseppina Mandalari, Maria Teresa Sciortino

**Affiliations:** 1 Department of Chemical, Biological, Pharmaceutical and Environmental Sciences, University of Messina, Viale Ferdinando Stagno d’Alcontres 31, 98166 Messina, Italy; paola.trischitta@studenti.unime.it (P.T.); maria.tamburello1@studenti.unime.it (M.P.T.); dbarreca@unime.it (D.B.); mtsciortino@unime.it (M.T.S.); 2 Department of Chemistry, Biology and Biotechnology, University of Perugia, Via Elce di Sotto 8, 06123 Perugia, Italy

**Keywords:** natural antivirals, HSV-1, zeaxanthin, ocular herpetic infection, extraction method

## Abstract

The search for alternative clinical treatments to fight resistance and find alternative antiviral treatments for the herpes simplex virus (HSV) is of great interest. Plants are rich sources of novel antiviral, pharmacologically active agents that provide several advantages, including reduced side effects, less resistance, low toxicity, and different mechanisms of action. In the present work, the antiviral activity of Californian natural raw (NRRE) and roasted unsalted (RURE) pistachio polyphenols-rich extracts was evaluated against HSV-1 using VERO cells. Two different extraction methods, with or without *n*-hexane, were used. Results showed that *n*-hexane-extracted NRRE and RURE exerted an antiviral effect against HSV-1, blocking virus binding on the cell surface, affecting viral DNA synthesis as well as accumulation of ICP0, UL42, and Us11 viral proteins. Additionally, the identification and quantification of phenolic compounds by RP-HPLC-DAD confirmed that extraction with *n*-hexane exclusively accumulated tocopherols, carotenoids, and xanthophylls. Amongst these, zeaxanthin exhibited strong antiviral activity against HSV-1 (CC_50_: 16.1 µM, EC_50_ 4.08 µM, SI 3.96), affecting both the viral attachment and penetration and viral DNA synthesis. Zeaxanthin is a dietary carotenoid that accumulates in the retina as a macular pigment. The use of pistachio extracts and derivates should be encouraged for the topical treatment of ocular herpetic infections.

## 1. Introduction

Herpes simplex type 1 (HSV-1) is an enveloped DNA virus belonging to the *Alphaherpesvirinae* subfamily. HSV-1 pathogenicity is associated with various clinical manifestations, including orofacial lesions, known as “cold sores”, keratitis, and conjunctivitis. Although HSV-1 infection generally does not lead to serious illness, it is responsible for ocular diseases or encephalitis in immunocompromised individuals, including transplanted or AIDS patients and newborns. The virion comprises three main structural elements: an icosahedral nucleocapsid containing a large genome of 152-kbp surrounded by a tegument layer consisting of viral proteins with multifunctional roles [1]. The outward envelope bilayer consists of a set of proteins required for attachment and entry, such as gD, gH, gL, gC and gB [2]. The infection begins with the interaction between the gD protein and HVEM (Herpesvirus Entry Mediator) host receptor, allowing tight anchoring on the cell surface. Upon receptor binding, gD changes structural conformation and interacts with pre-complexed gH/gL, which recruits gB to form the fusion pore and releases nucleocapsid and tegument proteins into the cytoplasm [3]. The viral DNA is transported and released into the nucleus and converted into a covalently closed circular molecule to start the viral replication and viral gene transcription. All viral genes are sequentially expressed, starting from the immediate early (IE) genes group, such as ICP0, ICP4, ICP22, ICP27, and ICP47, which are responsible for gene regulation, followed by the early α genes group, that are involved in the replication of the viral DNA. After these events, the late (L) genes group will encode structural proteins required for the assembly of the new progeny [4]. Primary HSV-1 infection occurs in epithelial cells of the oral, nasal, or ocular mucosa and produces a localized infection in the site of entry, generating skin vesicles or mucosal ulcers associated with the spread of the virus. The virus can also establish a permanent HSV infection in the host localizing in the dorsal root ganglia. Sporadic reactivation of HSV can occur spontaneously or in response to various stimuli, resulting in increased production of infectious virions, which move with an anterograde movement to the initial site of infection, where lytic replication occurs [5]. Primary herpes simplex ocular infections can cause mild inflammation of the cornea or more serious and permanent intraocular inflammations such as keratouveitis [6]. Herpetic keratouveitis generally follows a chronic course and represents a major cause of blindness worldwide [7]. Antiviral drugs targeting viral DNA replication, i.e., guanosine analogues, together with corticosteroids to mitigate the inflammatory processes, are used as therapeutic approaches during herpetic infection [8,9]. These synthetic drugs are associated with several side effects, and their prolonged use favors drug resistance. Therefore, the identification of novel antiherpetic molecules exhibiting low toxicity against drug-resistant HSV isolates is challenging. Phytochemical nutraceuticals are characterized by bioactive compounds that exhibit a specific action in human metabolic processes. Some of these, including polyphenols (flavonoids and non-flavonoids) and carotenoids, have an impact on ocular diseases [10,11,12,13]. Flavonoids are the main class of phenolic compounds present in pistachios (*Pistacia vera* L.), whereas the xanthophyll carotenoids (lutein and zeaxanthin) are responsible for providing the color to pistachio nuts [14]. Carotenoids have demonstrated a positive influence on cataracts and in other eye conditions through their antioxidant, anti-inflammatory, and photoprotective role [15,16,17]. Zeaxanthin accumulates in the retina as a macular pigment and has been shown to have a protective role in eyes quenching reactive oxygen species (ROS), which cause age-related macular degeneration [18,19,20]. Here, we investigated the role of Californian natural raw (NRRE) and roasted unsalted (RURE) pistachio polyphenols-rich extracts against HSV-1 replication, obtained by two different extraction methods. Amongst pure compounds, the antiviral effect of zeaxanthin and its mechanism of action was determined.

## 2. Materials and Methods

### 2.1. Plant Material and Extraction Methods

Californian natural shelled (NPs, Pistacchi Sgusciati California Pissgsu01/BSV1, L673-16221262) and roasted (RPs, Pistacchi Sgusciati Tostati California Pissgstu01/BSV1) pistachio kernels were kindly supplied by the American Pistachio Growers (Fresno, CA, USA). Polyphenolic extracts were prepared following two different extraction methods as previously reported [21]. Briefly, in the first method (extraction method 1), NPs or RPs (10 g) were extracted with *n*-hexane (100 mL) five times for 2 h, in order to separate the lipid fraction, and the residues were combined with methanol/HCl 0.1% (*v*/*v*) (100 mL).

In the second method (extraction method 2), NPs or RPs (10 g) were extracted with methanol/HCl 0.1% (*v*/*v*) (100 mL), and the methanol extracts were passed through a Solid phase extraction column (SPE, Supelclean™ LC-18 SPE cartridge) to obtain a flavonoid enriched fraction. All tested pistachio extracts were dissolved in DMSO at 100 mg/mL.

### 2.2. Polyphenolic Analysis of Pistachio Extracts

RP-HPLC-DAD identification and quantification of phenolic compounds was performed as previously reported [21,22]. Briefly, a Shimazu system, having an LC-10AD pump system, a vacuum degasser, a quaternary solvent mixing, an SPD-M10A diode array detector, and a Rheodyne 7725i injector, was used for the compound identification. Peak identity was verified by comparing their absorption spectra and retention times with those of pure (≥99%) commercial standards. Tocopherols and xanthophylls were quantified by HPLC, whereas β-carotene was quantified by ultraviolet-visible spectroscopy. HPLC separations were performed on a microsilica column (Ascentis Supelco SI; 250 × 1.0 mm, 5-μm particle size) with a mobile phase consisting of *n*-hexane/isopropanol (99:1). The flow rate was 1.0 mL/min, and the injection volume was 20 μL. The method was validated according to the Eurachem guidelines for each compound, namely α-tocopherol, γ-tocopherol, and δ-tocopherol [23]. The results were expressed as milligrams per 100 g. The β-carotene was quantified using a Varian Carry 50 ultraviolet-visible spectrophotometer at 450 nm utilizing a calibration curve prepared daily. Zeaxanthin was analyzed according to Bouali et al. 2013 with little modifications. For HPLC separations, a BioDiscovery C18 column analytical column (250 × 4.6 mm inner diameter, 5-mm particle size) was used. The mobile phase was a mixture of acetonitrile/methanol and 50 mM ammonium acetate/water/dichloromethane (700:150:50:100, *v*/*v*/*v*/*v*) at a flow rate of 1 mL/min. Samples (20 µL) were injected, the wavelengths set at 450 nm, and the spectra recorded between 220 and 600 nm. Identification was carried out by comparing the retention times and absorption spectra of unknown peaks to authentic standards of zeaxanthin (purity > 95%). Quantification was based on the external standard method. Results were expressed as milligrams per 100 g. For biological analysis, the tocopherols were dissolved in ethanol at 1 M, β-carotene and luteolin in DMSO at 2Mm, and zeaxanthin in DMSO at 500 µM. 

### 2.3. Cell Culture and Virus

VERO cell lines (American Type Culture Collection) were propagated in minimal essential medium (EMEM) and supplemented with 6% fetal bovine serum (FBS) (Lonza, Belgium) at 37 °C under 5% CO_2_. The prototype HSV-1 (F) strain was kindly provided by Dr. Bernard Roizman (University of Chicago, Chicago, IL, USA), and the virus stock was produced and titered in VERO cells.

### 2.4. Cell Viability Assay

To assess the cell viability following pistachio extract treatment, the ViaLight™ Plus Cell Proliferation and Cytotoxicity Bioassay (Lonza Group Ltd., Basel, Switzerland) was employed. Vero cells were grown in wells of 96-well plates and treated with different concentrations of extracts (0.4, 0.6, 0.8, 1.2, and 1.6 mg/mL) for 72 h. The GloMax^®^ Multi Microplate Luminometer (Promega Corporation, 2800Woods Hollow Road, Madison, WI, USA) in combination with the ViaLight™ plus cell proliferation and cytotoxicity bioassay kit quantified the emitted light intensity related to ATP degradation. The measured luminescence value was converted to the cell proliferation index (%) according to the following equation:

Cell viability% = (A − B)/(C − B)

(where A denotes the average of treated samples, B represents background luminescence, and C represents the average of untreated samples).

A CCK-8 assay (ab228554; Abcam, Cambridge, UK) was performed to evaluate the cytotoxicity of pure compounds. WST-8/CCK8 tetrazolium salt is reduced by cellular dehydrogenases to form an orange formazan product that is soluble in a tissue culture medium. The amount of formazan produced is directly proportional to the number of living and metabolically active cells and is measured via absorbance at 460 nm. Therefore, VERO cells (2 × 10^4^ cells/mL) were grown in 96-well microtiter plates at 37 °C in a 5% CO_2_ incubator for 24 h. Then, they were exposed to serial dilutions of pure compounds (β-carotene, zeaxanthin, luteolin, α, β, and δ tocopherol) for 72 h and incubated with CCK8 tetrazolium salt for 4 h at 37 °C in a CO_2_ incubator. The absorbance was measured at 460 nm using a GloMax^®^ Discover Microplate Reader (Promega, Madison, WI, USA), and the percentage of cellular viability was calculated and compared to untreated cells.

### 2.5. Plaque Reduction Assay 

VERO cells were pretreated with 0.3 mg/mL, 0.4 mg/mL, and 0.6 mg/mL of either NRRE or RURE and either NRRE *n*-hexane or RURE *n*-hexane and infected with pretreated HSV-1 dilutions for 1 h. Similarly, the cells were pre-treated with various concentrations of β-carotene, zeaxanthin, luteolin, α, β, and δ tocopherol, and infected with pre-treated HSV-1 dilutions for 1 h. After 1 h, unabsorbed viral dilutions were aspirated, and the monolayers were covered with a medium containing 0.8% methylcellulose in the presence of both extracts and pure compounds at different concentrations. DMSO was included as a control and used at the highest concentration (0.6 mg/mL). The plaques were visualized after 3 days using crystal violet staining and visualized with an inverted microscope for plaque morphology detection. Furthermore, the viral plaque’s size was measured using the ImageJ program. The data reported are means of at least 10 plaques for wells. Photographs of individual plaques were reported.

### 2.6. Binding Assay

The binding assay was performed at a temperature of 4 °C, which permits viral binding but not entry [24]. Thus, the only mechanism that a potential inhibitor can disrupt in this assay is virus binding to the cell. The viral suspension was incubated with 0.3 mg/mL, 0.4mg/mL, and 0.6 mg/mL of NRRE *n*-hexane or RURE *n*-hexane for 1 h prior to assay and incubated on ice. VERO cells were plated in 6-well plates and allowed to reach confluence whereupon the plates were then removed from the incubator and left at room temperature for about 15 min, and subsequently incubated at 4 °C. The cold inoculum containing different concentrations of pistachio extracts in the presence of a virus or control virus was used to infect the cells. Plates were then incubated at 4 °C for 1 h to allow the virus to bind to the cells. The unbound viruses were removed by rinsing the plates with cold PBS three times. Then, plates were overlaid with a complete medium containing 0.8% methylcellulose and incubated at 37 °C for three days. The crystal violet staining allows for the visualization of the viral plaques with the inverted microscope.

### 2.7. The Time-of-Addition Assay 

Vero cells were subjected to three different experimental conditions, namely (1) cell pretreatment, (2) virus pretreatment, and (3) post-adsorption treatment. (1) Cell-pretreatment: Vero monolayer cells were pretreated with zeaxanthin at 37 °C for 1 h, after which the medium was replaced with HSV-1 (MOI 1). (2) Virus pretreatment: HSV-1 was pretreated with zeaxanthin at 4 °C for 1 h and then used to infect the VERO monolayers for a further 1 h. (c) Post-adsorption treatment: VERO cells were infected at 37 °C with HSV-1 for 1 h, and zeaxanthin was added after adsorption. In each experimental condition, zeaxanthin (10 µM) was used, and the monolayers were incubated with a medium containing 0.8% methylcellulose in order to visualize the viral plaques after 3 days using crystal violet staining. The infected samples were harvested 24 h post-infection and subjected to viral DNA extraction.

### 2.8. Viral Inactivation Assay

A viral inactivation assay was performed as previously described with some modifications [25]. HSV-1 (10^4^ PFU/mL) was mixed with NRRE *n*-hexane, RURE *n*-hexane, and zeaxanthin at EC_50_ values reported in Table 1 and then incubated at 37 °C for 1 h. The test extracts–virus mixture was then diluted 100-fold (final virus concentration, 100 PFU/well) with DMEM containing 1% FBS, and the virus inoculum was subsequently added to monolayers of Vero cells seeded in 12-well plates. The untreated HSV-1 sample was used under the same conditions. After adsorption for 1 h at 37 °C, the diluted inoculum was discarded, and the cells were washed with PBS twice and incubated with a medium containing 0.8% methylcellulose to visualize the viral plaques after 3 days using crystal violet staining.

### 2.9. Western Blot Analysis

Immunoblot analysis was carried out to evaluate the accumulation of viral proteins as previously reported [26]. Briefly, total cells lysates were prepared from cells by SDS sample buffer 1X (62.5 mM Tris-HCl (Tris(hydroxymethyl) aminomethane hydrochloride) pH 6.8; 50 mM DTT (dithiothreitol); 10% glycerol; 2% SDS (sodium dodecyl sulfate); 0.01% Bromophenol Blue; EDTA-free Protease Inhibitor Cocktail 1X (Roche)), and after they are boiled for 5 min. An equal amount of protein extract was loaded onto a 10% sodium dodecyl sulfate-polyacrylamide gel, transferred to nitrocellulose membranes, and blocked at 4 °C overnight in 5% non-fat dry milk-TBS. GAPDH (sc-32233), UL42 (sc-53333), ICP0 (sc-56985) (Santa Cruz, CA, USA), and US11 (provided by Professor Bernard Roizman) were detected by secondary HRP conjugated goat anti-mouse IgG (Merk, Millipore). Specific bands were visualized using Immobilon Classico Western HRP substrate (Merk, Millipore). Quantitative densitometry analysis of immunoblot band intensities was performed using ImageJ software. The intensity of the target protein was divided by the intensity of the GAPDH and graphically represented by GraphPad Prism 6 software (GraphPad Software, San Diego, CA, USA).

### 2.10. Viral DNA Extraction and Real-Time PCR

The viral DNA was extracted using TRIzol^®^ (Thermo Fisher Scientific, Waltham, MA, USA) according to the manufacturer’s instructions. 

The procedure, based on the phenol/chloroform extraction method, allows for the precipitation of viral DNA from the organic phase [26]. The DNA pellet was washed twice in a solution containing 0.1 M trisodium citrate in 10% ethanol and then dissolved in 8 mM NaOH. The concentration of DNA was determined by fluorometer analysis with the Qubit double-stranded DNA (dsDNA) HS (High Sensitivity) Assay Kit according to the manufacturer’s instructions. The amplification of viral DNA was carried out by TaqMan™Universal Master Mix II (Applied Biosystems™, Foster City, CA, USA) in a 50 µL reaction mixture containing the following: TaqMan Universal Master Mix II, DNA (100 ng); HSV-1 forward (10 µM) and HSV-1 reverse (10 µM) primers (Fw 5′-catcaccgacccggagagggac-’3; Rev 5′-gggccaggcgcttgttggtgta-’3); and the TaqMan probe (5 µM) (5′-6FAM-ccgccgaactgagcagacacccgcgc-TAMRA-’3, where 6FAM is 6-carboxyfluorescein and TAMRA is 6-carboxytetramethylrhodamine). The amplification was carried out on Applied Biosystems 7300 Real-Time PCR System under the following conditions: 10 min at 95 °C, 60 s at 95 °C for 40 cycles, 30 s at 60 °C, and 30 s at 72 °C. Each amplification run contained one negative control. The primers were designed on a large catalytic subunit of HSV-1 DNA polymerase holoenzyme (UL30 gene). The relative quantitation of HSV-1 DNA was generated by the comparative Ct method using GAPDH as a housekeeping gene (GAPDH forward 5′-GAGAAGGCTGGGGCTCAT-3′ and reverse 5′- TGCTGATGATCTTGAGGCTG-3′). One-way analysis of variance (ANOVA) and the GraphPad Prism 6 software (GraphPad Software, San Diego, CA, USA) was used to perform statistical analysis and graphical representations, respectively.

### 2.11. Statistical Analysis

Three independent experiments were carried out in triplicate (n = 3) for each assay, and the results represent the average +/− SD. The statistical analysis was performed with GraphPad Prism 8 software (Graph-Pad Software, San Diego, CA, USA) using one-way variance analysis (ANOVA). The significance of the *p*-value is indicated with asterisks (*, **, ***, ****), denoting the *p*-value significance levels less than 0.05, 0.01, 0.001, and 0.0001, respectively. The half-maximal cytotoxic concentration (CC_50_) and the half-maximal effective concentration (EC_50_) values were calculated using non-linear regression analysis.

## 3. Results

### 3.1. RP-HPLC-DAD Identification and Quantification of Phenolic Compounds

As previously reported [21], the major identified compounds were gallic acid, catechin, cyanidin-3-O-galactoside, and isoquercetin. Extraction method 2 resulted in an increase (~1.3-fold) in the total amount of phenolic compounds compared with extraction method 1. 

### 3.2. Evaluation of the Cytotoxicity Effect Following Treatment with Pistachio Extracts

**Figure 1:**
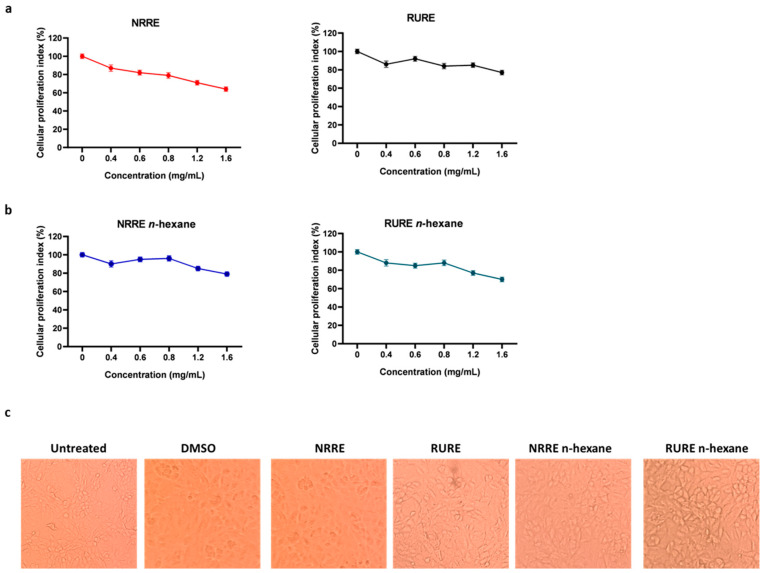
Cells viability of VERO cells treated with NRRE and/or RURE extracted with or without *n*-hexane. VERO cells were incubated with 0.4, 0.6, 0.8, 1.2, and 1.6 mg/mL of extracts for 72 h. Results represent the mean of three biologically independent experiments ± SD. (**a**) Californian natural raw pistachio polyphenols-rich extracts (NRRE) and Californian roasted unsalted (RURE) pistachio polyphenols-rich extracts; (**b**) *n*-hexane-extracted Californian natural raw pistachio polyphenols-rich extracts (NRRE *n*-hexane) and *n*-hexane-extracted Californian roasted raw pistachio polyphenols-rich extracts (RURE *n*-hexane); (**c**) micrographs of Vero monolayer at 10× magnification after 72 h treatment with 0.6 mg/mL of NRRE, RURE, NRRE *n*-hexane, and RURE *n*-hexane.

The cytotoxic effect of pistachio kernels was evaluated by monitoring the metabolic activity of cells following natural raw polyphenols-rich extract (NRRE) and/or roasted unsalted polyphenols-rich extract (RURE) treatment. The dose-dependent cytotoxicity of both extracts was assayed on VERO cells. Both extracts did not show a marked cytotoxic effect except for exposition > to 0.8 mg/mL, which moderately affected cellular proliferation (Figure 1a,b). Lower concentrations did not show cytotoxicity and did not alter the cell morphology (Figure 1c). Based on the above results and on the CC_50_ values reported in Table 1, we used concentrations up to 0.6 mg/mL for further experiments. 

### 3.3. Impact of NRRE and RURE with or without n-Hexane on HSV-1 Replication in VERO Cells

**Figure 2:**
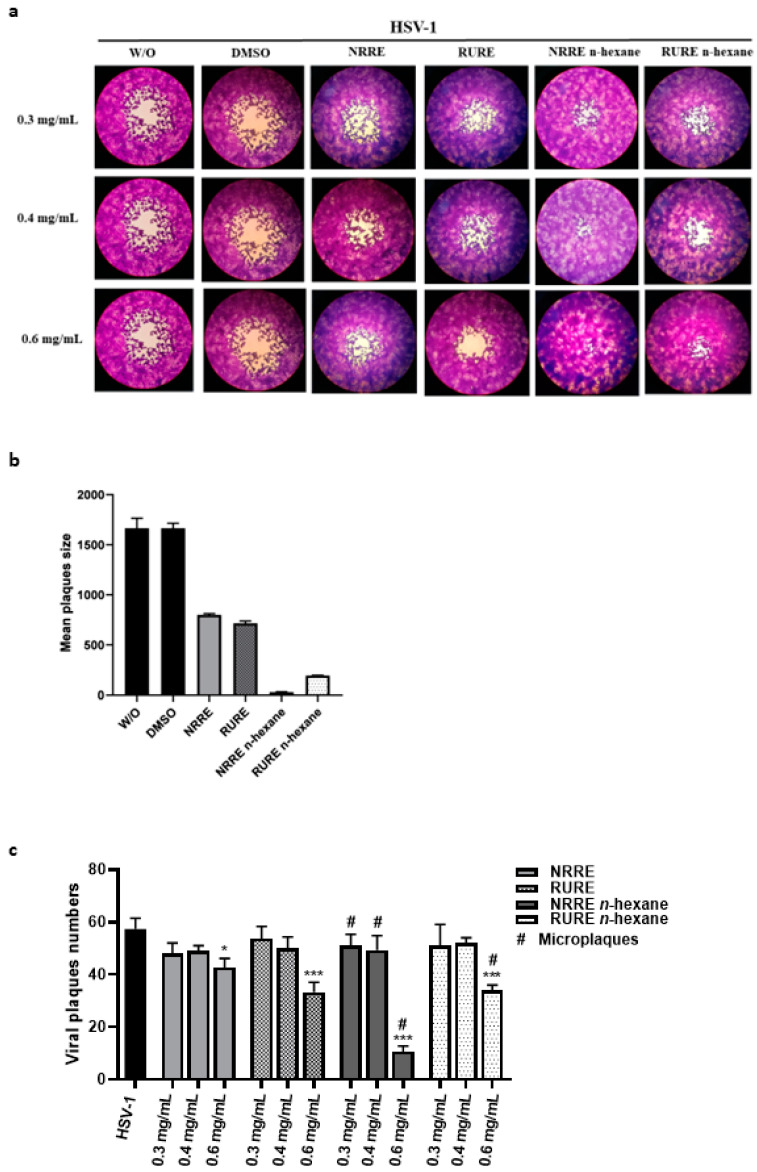
Plaque reduction assay. VERO cells were pre-treated with 0.3 mg/mL, 0.4mg/mL, and 0.6 mg/mL of either NRRE or RURE and either NRRE *n*-hexane or RURE *n*-hexane and infected with pre-treated HSV-1 dilutions for 1 h. After 1 h, unabsorbed viral dilutions were aspirated, and the monolayer was covered with medium containing 0.8% methylcellulose in the presence of NRRE, RURE, NRRE *n*-hexane, or RURE *n*-hexane at different concentrations (0.3 mg/mL, 0.4 mg/mL, and 0.6 mg/mL) and solvent DMSO. The plaques were stained after 3 days using crystal violet staining and visualized with an inverted microscope (Leica DMIL, Nuβloch, Germany) for plaque morphology detection. In panel (**a**), the images show the plaque morphological changes, in panel (**b**) the area of viral plaque size (at 0.6 mg/mL) was measured using ImageJ program and reported in mm^2^, whereas panel (**c**) reports the statistical analysis of the plaque’s reduction assay. The data were analysed as the means of triplicates ± SD for each dilution. # indicates microplaques detection and asterisks (* and ***) indicate the significance of *p*-values less than 0.1 and 0.001, respectively.

Based on the results obtained in the cytotoxicity assay, the antiviral activity was evaluated on serial non-cytotoxic dilutions of the extracts, starting from 0.3 to 0.6 mg/mL, by plaques reduction assay (Figure 2a–c). VERO cells and viral dilutions were simultaneously pre-treated with either NRRE or RURE and either NRRE *n*-hexane or RURE *n*-hexane at non-cytotoxic concentrations (0.3 mg/mL, 0.4 mg/mL, and 0.6 mg/mL) for 1 h and then mixed to allow viral adsorption. After 1 h, any unabsorbed virus was aspirated and the monolayer was covered with Dulbecco’s Modified Eagle’s Medium containing 0.8% methylcellulose in the presence of both extracts, separately. Both NRRE and RURE exhibited a significant inhibitory activity at 0.6 mg/mL, and in particular, the mixture extracted with *n*-hexane determined a significant reduction of plaque numbers (Figure 2c) and sizes, as reported by the change of morphology and by microplaques detection (Figure 2a,b). The selectivity index (SI) was determined by the ratio of CC_50_ to EC_50_ (Table 1). 

### 3.4. Mechanisms Involved in the Anti-HSV-1 Exerted by NRRE and RURE n-Hexane

**Figure 3:**
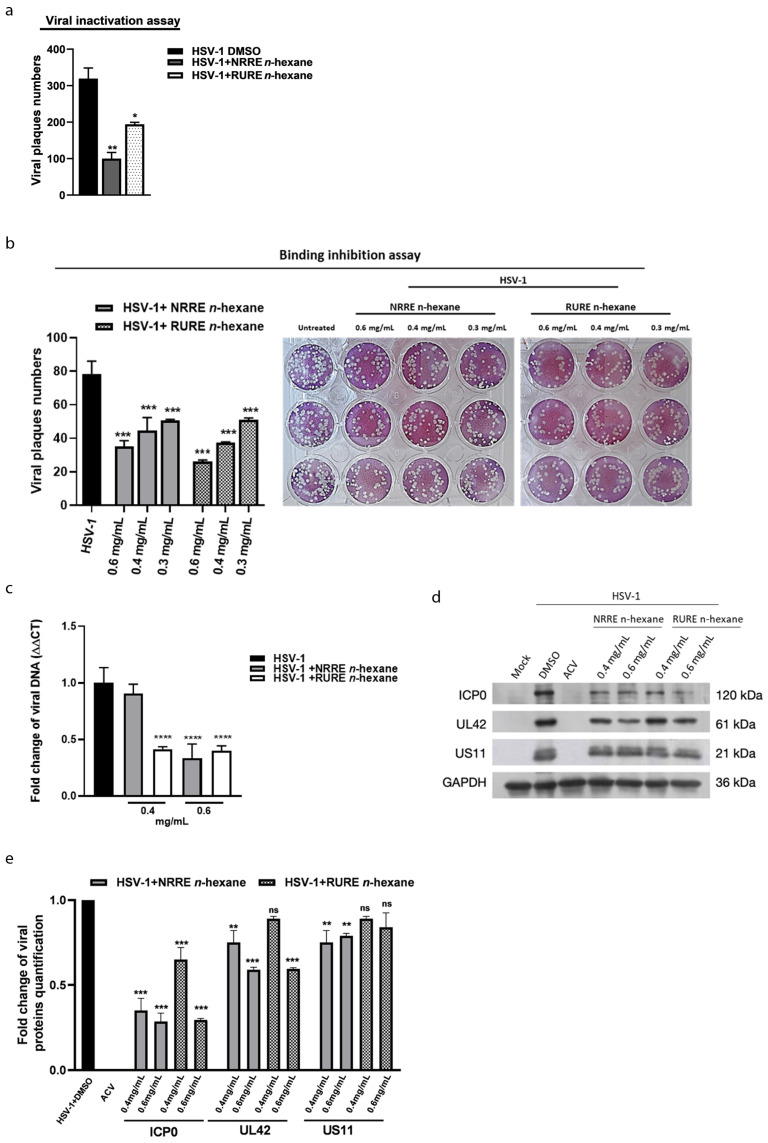
Characterization of the antiviral mechanism mediated by NRRE *n*-hexane or RURE *n*-hexane. (**a**) Viral inactivation assay. HSV-1 (10^4^ PFU/mL) was mixed with NRRE *n*-hexane and RURE *n*-hexane at EC_50_ values and then incubated at 37 °C for 1 h. The procedure is reported in materials and methods. (**b**) Binding inhibition assay of HSV-1. VERO cells were infected with HSV-1 and incubated with 0.3 mg/mL, 0.4 mg/mL, and 0.6 mg/mL of both NRRE *n*-hexane or RURE *n*-hexane. The binding was carried out for 1 h at 4 °C. After 1 h, unabsorbed viral particles were removed, and the monolayer was washed with cold PBS and covered with methylcellulose. The right side of panel (**a**) show a representative experiment of plaques assay used for the binding. In panels (**c**–**e**), VERO cells and HSV-1 (MOI 1) were incubated with 0.4 mg/mL and 0.6 mg/mL of either NRRE *n*-hexane or RURE *n*-hexane for 24 h as described in material and methods. DMSO was used as control sample. Samples were used for viral DNA quantification (**c**) and viral proteins analysis (**d**,**e**). GAPDH was used as a housekeeping gene. Panel (**e**) shows fold changes in viral protein quantification. Data are expressed as a mean (±SD) of at least three experiments and asterisks (*, **, ***, ****) indicate the significance of *p*-values less than 0.05, 0.01, 0.001, and 0.0001, respectively.

Based on the antiviral activity displayed by NRRE and RURE *n*-hexane, we aimed to evaluate the mechanism involved in the inhibition of HSV-1 replication. Thus, we tested the effect of both extracts (i) on the inactivation of HSV-1 (Figure 3a); (ii) during the binding of the virus on the cells surface (Figure 3b); (iii) during the viral DNA replication (Figure 3c); and (iv) on viral proteins synthesis (Figure 3d,e). To evaluate the antiviral mechanism mediated by the extracts, we investigated their effect on the virus particles using the viral inactivation assay. We found a marked inhibition of both extracts when pre-incubated for 1 h with the virions (Figure 3a), suggesting that both extracts can bind to virus particles and neutralize virus infectivity.

Thus, to test whether the binding with virions affects attachment and penetration, HSV-1 suspension, in the presence of different concentrations of NRRE and RURE *n*-hexane (0.3 mg/mL, 0.4 mg/mL, and 0.6 mg/mL), was used to infect VERO cells for 1 h at 4 °C. The low temperature allows for binding but not the penetration of the virions inside the cells. The results, reported in Figure 3b, show that NRRE and RURE *n*-hexane exhibited significant inhibitory activity on the virus binding mainly at 0.6 mg/mL. However, the treatments were unable to completely block viral replication. Images of VERO cells infected and treated with various concentrations of extracts were reported on the right side of Figure 3b.

To determine if the treatment with NRRE and RURE *n*-hexane interfered with the production of the viral genome, real-time PCR was used to compare the relative levels of total viral DNA produced during viral replication in the presence of NRRE and RURE *n*-hexane compared with the untreated HSV-1 (Figure 3c). The results show that both extracts (0.6 mg/mL) reduced viral DNA accumulation by 50%. Similarly, a reduction in viral protein accumulation of ICP0, UL42, and Us11, which are representative products of the viral gene cascade (α, β, and γ, respectively) (Figure 3d,e) was detected. We conclude that cell treatment with NRRE and RURE *n*-hexane does not support HSV-1 productive infection.

### 3.5. Determination of Anti-HSV Activity Properties of Tocopherols, Carotenoids, and Xanthophylls Exclusively Present in n-Hexane Pistachios Extraction Method

**Figure 4:**
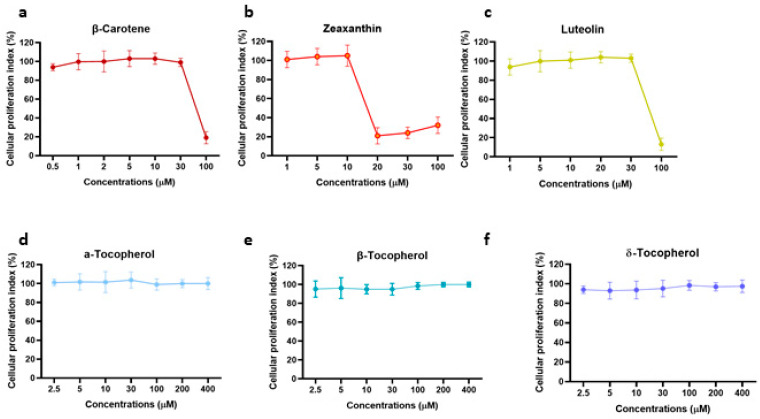
Cell viability of representative pure compounds present in NRRE and RURE *n*-hexane. (**a**–**f**) VERO cells were incubated with different concentrations of β-carotene, zeaxanthin, luteolin, α, β, and δ tocopherol for 72 h. The absorbance was measured at 460 nm, and the % of cell viability was calculated with respect to the untreated cells. Data are expressed as a mean (±SD) of at least three experiments.

**Figure 5:**
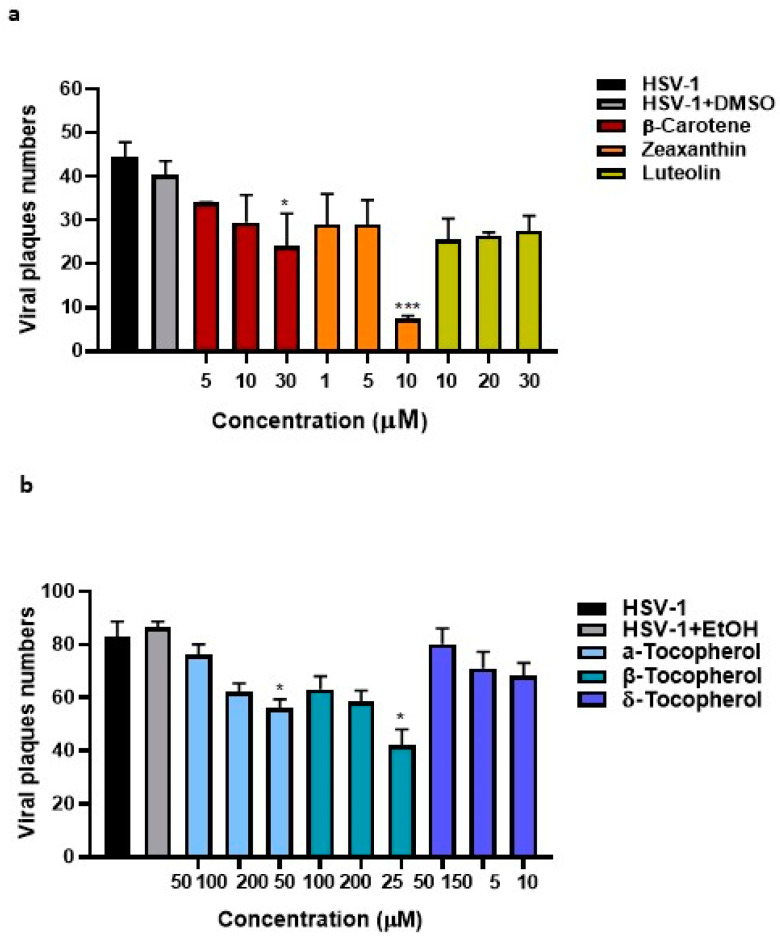
Plaque reduction assay following β-carotene, zeaxanthin, luteolin, α, β, and δ tocopherol treatment. VERO cells were pre-treated with various concentrations of β-carotene, zeaxanthin, luteolin, α, β, and δ tocopherol and infected with pre-treated HSV-1 dilutions for 1 h. After 1 h, the monolayer was covered with a medium containing 0.8% methylcellulose in the presence of single compounds. DMSO and ethanol (EtOH) were used as solvent control. The plaques were visualized after 3 days using crystal violet staining and visualized with an inverted microscope (Leica DMIL, Nuβloch, Germany) for plaque morphology detection. Panel (**a**) reports the plaque reduction assay following β-carotene, zeaxanthin, and luteolin treatment. Panel (**b**) reports the reduction of viral plaques following α, β, and δ tocopherol. Results are expressed as mean ± standard deviation (n = 3). * < 0.05 and *** < 0.001.

The data reported above showed that the pistachio extracts obtained by *n*-hexane extraction inhibited viral replication. In order to understand whether certain pure compounds, exclusively present in pistachios after *n*-hexane extraction, were responsible for the blockage of viral replication, we tested the antiviral activity of tocopherols, carotenoids, and xanthophylls. Our choice was supported by the phytochemical characterization (Table 2) in which α, β, and δ tocopherol, β-carotene, and zeaxanthin were amongst the most abundant compounds present in NRRE *n*-hexane and RURE *n*-hexane and completely absent in NRRE and RURE. Luteolin was chosen since it was present in greater quantities using extraction method 2 [21]. Before exploring any antiviral activity, the viability assay was carried out by treating VERO cells with different concentrations of pure compounds to determine the non-toxic concentration for further experiments (Figure 4). Accordingly, the antiviral activity was evaluated following treatment with non-cytotoxic dilutions of the β-carotene, zeaxanthin, luteolin, α, β, and δ tocopherol (Figure 5a,b). Our results report a significant reduction in viral plaques following treatment with zeaxanthin at a concentration of 10µM. On the other hand, the other employed compounds did not exhibit any significant inhibitory effect on HSV-1.

### 3.6. Zeaxanthin Inhibits the HSV-1 Replication Acting on Both Viral Internalization and Replication

**Figure 6:**
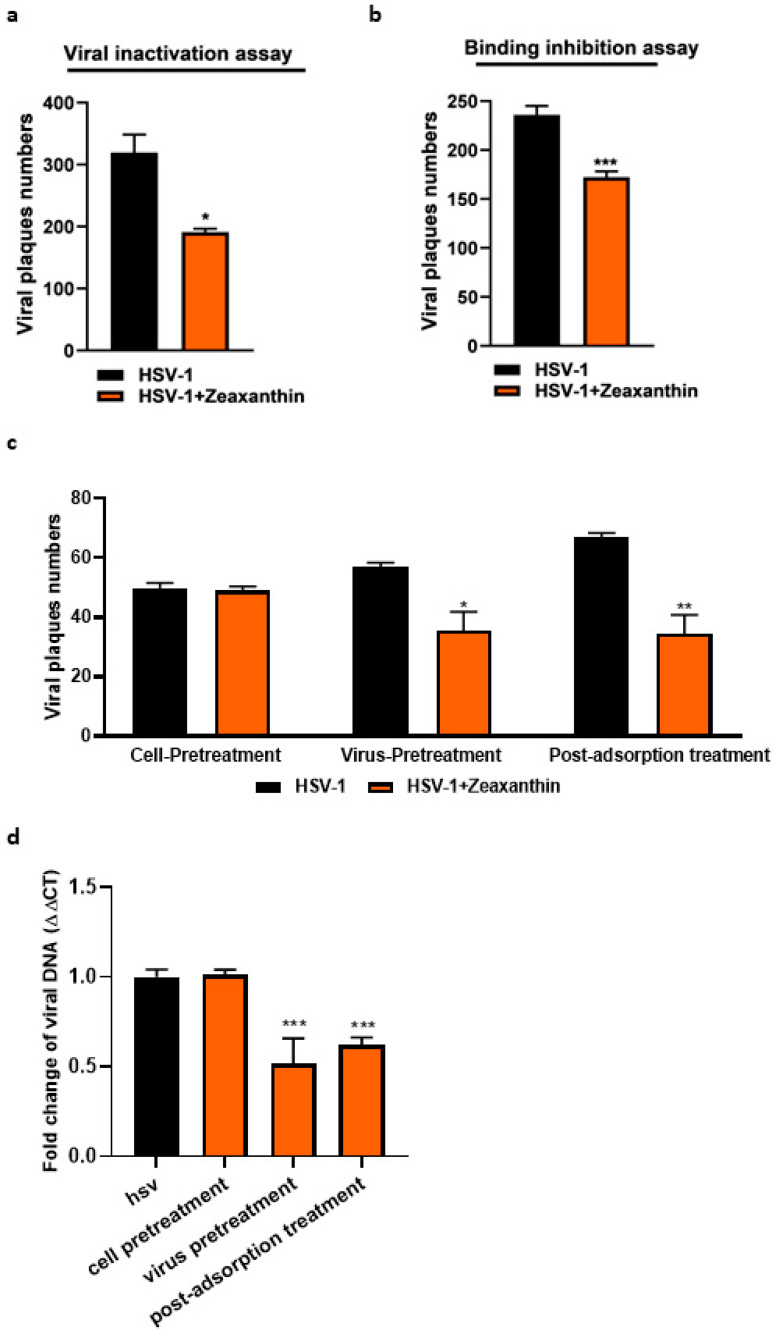
Evaluation of zeaxanthin on HSV-1 replication. (**a**) Viral inactivation assay. HSV-1 was mixed with NRRE *n*-hexane and RURE *n*-hexane at EC50 values and then incubated at 37 °C for 1 h. The procedure is reported in the materials and methods. (**b**) Binding inhibition assay of HSV-1 infection. VERO cells were infected with a zeaxanthin-pre-treated virus for 1 h at 4 °C. After 1 h, the monolayer was washed with cold PBS and covered with a medium containing methylcellulose. (**c**) VERO cells were subjected to three different experimental conditions as follows: (1) cell pretreatment, (2) virus pretreatment, and (3) treatment of infected cells post adsorption in the presence or not of zeaxanthin (10 μM). The plaques were visualized after 3 days. (**d**) Samples for viral DNA determination were treated as described before harvested and 24 h post-infection for real-time PCR analysis. The HSV-1 infection was carried out at MOI 1. Data are expressed as a mean (±SD) of at least three experiments. * *p* < 0.05, ** *p* < 0.01, and *** < 0.001 vs. HSV-1.

To elucidate the antiviral mechanism exerted by zeaxanthin, different experimental procedures were performed; (i) first, viral inactivation assay (Figure 6a): we found a marked inhibition of zeaxanthin when directedly pre-incubated for 1 h with the virions, suggesting that it could bind to virus particles and neutralize virus infectivity; (ii) second, the binding assay (Figure 6b): results indicated that the zeaxanthin did not totally prevent viral entry, suggesting an additional antiviral mechanism downstream of viral entry; (iii) lastly, different treatment conditions were performed to determine the stage(s) of viral replication at which zeaxanthin exerted its inhibitory activity. Thus, zeaxanthin was used to treat the cells, to treat the viral suspension, and to treat cells after viral adsorption. After 24 h, the samples were collected and processed to determine viral titers (Figure 6c) and viral DNA synthesis (Figure 6d). As shown in Figure 6c, pretreatment of HSV-1 suspension with 10 μM zeaxanthin significantly reduced viral titer, compared to the untreated virus control (*p* < 0.05), suggesting that zeaxanthin may have had direct interaction with HSV-1 viral particles. On the other hand, cell treatment with zeaxanthin, prior to infection, did not block viral adsorption. Treatment of infected cells with zeaxanthin after virus adsorption affected viral titer (*p* < 0.001). Similar results were obtained by analyzing viral DNA synthesis in the three different experimental conditions (Figure 6d). Therefore, we confirm that zeaxanthin interacts with virus particles by blocking HSV-1 binding and can inhibit viral replication following its release inside the cell.

## 4. Discussion

HSV-1 infection causes a wide variety of disease states, including orolabial herpes, herpetic folliculitis, ocular infection, herpes encephalitis, eczema, and severe or chronic HSV infection, usually treated with antivirals [27]. Indeed, anti-HSV drugs such as acyclovir, penciclovir, and ganciclovir, selectively inhibit the replication of HSV-1 acting as a substrate for viral DNA polymerase [28]. The prolonged use of guanosine analogues is associated with drug resistance and helps develop HSV drug-resistant strains, which represent a serious public health problem, especially for immunocompromised patients [29]. Therefore, the discovery of novel therapeutic approaches is a major challenge to fight herpetic infections. In this paper, we examined the effect of NRRE and RURE pistachio polyphenols-rich extracts against HSV-1 infection obtained by two different extraction methods. Consistent with previous results on the antibacterial activity [21], here we report a different antiviral effect due to the two extraction methods. Indeed, the polyphenol-rich fractions of NRRE and RURE extracted with *n*-hexane (extraction method 1) resulted in more activity against HSV-1 replication than NRRE and RURE extracts obtained with extraction method 2 (Figure 2).

The effects of solvents and extraction methods on the polyphenolic contents and biological activities of plant and fruit extracts have been previously reported [30]. The solvents used for the extraction are known to impact the yield and qualitative profile of the polyphenols, and, therefore, on their biological activities [31].

The drying process also influences the polyphenolic content and could be responsible for the lower content of RPs [21,32]. In line with previous results [21,33,34], the natural extracts were more active against HSV-1 compared with the roasted. The synergistic interactions amongst the polyphenolic compounds in the natural and roasted extracts are thought to play a key role in the antiviral effect.

We have recently reviewed the production, nutrients, phytochemical composition, and emerging research on the health benefits of pistachios [14]. Pistachios are a good source of nutrients and phytochemicals such as protein, fiber, monounsaturated fatty acids, minerals and vitamins, carotenoids, phenolic acids, flavonoids, and anthocyanins. Particularly, carotenoids are reported to protect the retina, and thus vision, as antioxidants and by acting as a blue light filter [35]. Based on this evidence and knowing that HSV-1 is responsible for eye maculopathies, we assessed whether certain pure compounds, exclusively present in pistachios after *n*-hexane extraction, could be responsible for the blockage of viral replication and tested the antiviral activity of some tocopherols, carotenoids, and xanthophylls. A recent review has highlighted the promising roles of carotenoids as treatments against emerging diseases and related symptoms caused by viral infections, including COVID-19 [17]. A variety of active phytochemicals, including flavonoids, terpenoids, organosulfur compounds, limonoids, lignans, sulphides, polyphenolics, coumarins, saponins, chlorophyllins, furyl compounds, alkaloids, polyines, thiophenes, proteins, and peptides have shown therapeutic applications against different viruses [36]. However, to our knowledge, no reports on the antiviral activity of zeaxanthin against HSV-1 have been reported. We have demonstrated a significant reduction of HSV-1 replication following treatment with zeaxanthin (CC_50_ 16.1, EC_50_ 4.08 µM, SI 3.96). Zeaxanthin is one of the most common carotenoids found in high quantities in green leafy vegetables but, unlike other carotenoids such as β-carotene, zeaxanthin does not contain vitamin A activity, and its antioxidant and anti-inflammatory properties contribute to a wide variety of health benefits [17,37,38,39,40,41]. Amongst carotenoids found in human blood and tissues, lutein and zeaxanthin are the only pigments found in the lens and in the central region of the retina and are involved in photoprotection from blue light cytotoxicity and ROS [42]. The exposure of blue light causes oxidative stress which damages the retina and leads to the development of glaucoma and keratitis [43,44]. In vitro studies reported a significant reduction of oxidative stress in the human lens’ epithelial cells following treatment with 5 µM of zeaxanthin for 48 h [45]. Similarly, in rat models, a diet including 40 mg of zeaxanthin allowed a reduction in proinflammatory cytokines in the retina [46]. The herpetic infections by the herpes simplex virus, varicella-zoster virus, and cytomegalovirus provoke acute or recurrent intraocular inflammations [47]. Ocular infection with HSV can cause inflammation of the eyelids, conjunctivae, iris, retina, and cornea [48]. HSV epithelial keratitis tends to resolve spontaneously within 1–2 weeks, while stromal keratitis is more likely to result in corneal scarring and loss of vision [49]. Therefore, the protective role of zeaxanthin in the eye could reduce the inflammatory processes due to recurrent herpetic infections.

The present study is the first to report on the antiviral activity of zeaxanthin and to define its molecular mechanism affecting HSV-1 replication. Interestingly, our results suggest that zeaxanthin specifically targeted HSV-1 viral particles by preventing their absorption into the cell membranes. These features make zeaxanthin a potential HSV-1 entry inhibitor. Our previous studies explored the antiviral potential of pistachio polyphenols against HSV-1, showing that catechin, eriodictyol-7-*O*-glucoside, gallic acid, protocatechuic acid, caffeic acid, rutin, and isoquercetin, the most abundant compounds identified, quantified, and extracted from natural shelled (NP) pistachio kernels, reduced viral DNA synthesis as well as the expression of viral proteins [49]. Indeed, flavonoids represent the main class of phenolic compounds in pistachios, whereas the xanthophyll carotenoids (lutein and zeaxanthin) are responsible for providing the color to pistachio nuts [14,22,34,49,50]. Zeaxanthin, exclusively present in pistachios after *n*-hexane extraction, inhibited HSV-1 replication acting both on viral binding and replication. 

## 5. Conclusions

This paper demonstrated that pistachio extracts exert an antiviral effect on HSV-1, acting on virus binding to the cell surface, viral DNA synthesis, and accumulation of viral proteins. Certain bioactives in pistachios, such as zeaxanthin, could be responsible for their antiviral potential. Given that humans are unable to synthesize zeaxanthin, its daily intake from dietary sources could help decrease virally induced inflammation. Lastly, topical use of zeaxanthin could be useful for protecting the eyes against herpetic infection.

## Figures and Tables

**Table 1:** Selectivity index (SI), cytotoxicity (CC_50_), and antiviral activity (EC_50_) of pistachio extracts.

Extract	CC_50_ (mg/mL)	EC_50_ (mg/mL)	SI
NRRE *n*-hexane	4.6	0.50	9.2
RURE *n*-hexane	3.29	0.66	4.98
NRRE	2.5	0.77	3.2
RURE	4.5	0.65	6.9

CC_50_: half maximal cytotoxic concentration; EC_50_: half maximal effective concentration; SI: selectivity index: the ratio of EC_50_/CC_50_.

**Table 2:** Identification and quantification of tocopherols, carotenoids, and xanthophylls in pistachio extracts. Values are expressed as mean ±SD (n = 3).

	mg/100 g			
Compound	RURE *n*-Hexane	NRRE *n*-Hexane	RURE	NRRE
	Extraction Method 1		Extraction Method 2	
*Tocopherols*				
a-tocopherol	trace	trace	-	-
β-tocopherol	0.17 ± 0.02	0.12 ± 0.02	-	-
δ-tocopherol	0.92 ± 0.08	0.62 ± 0.08	-	-
*Carotenoids*				
β-carotene	0.10 ± 0.01	0.11 ± 0.01	-	-
*Xanthophylls*				
Zeaxanthin	0.08 ± 0.01	0.07 ± 0.01	-	-

## Data Availability

The data presented in this study are available on request from the corresponding author.
